# Label-Free DNA Hybridization Detection Using a Highly Sensitive Fiber Microcavity Biosensor

**DOI:** 10.3390/s24010278

**Published:** 2024-01-03

**Authors:** Yao Wu, Guiyu Wang, Xiujuan Yu, Yuanji Fan, Xuefeng Chen, Shengchun Liu

**Affiliations:** 1 College of Physical Science and Technology, Heilongjiang University, Harbin 150080, China; 2211407@s.hlju.edu.cn (Y.W.); 2211411@s.hlju.edu.cn (G.W.); 2211409@s.hlju.edu.cn (Y.F.); 2 Heilongjiang Provincial Key Laboratory of Metamaterials Physics and Device, Heilongjiang University, Harbin 150080, China; 2003195@hlju.edu.cn (X.C.); liushengchun@hlju.edu.cn (S.L.)

**Keywords:** optical fiber sensor, Mach–Zehnder interferometer, DNA hybridization detection, label-free biosensor, open cavity

## Abstract

A novel label-free optical fiber biosensor, based on a microcavity fiber Mach–Zehnder interferometer, was developed and practically demonstrated for DNA detection. The biosensor was fabricated using offset splicing standard communication single-mode fibers (SMFs). The light path of the sensor was influenced by the liquid sample in the offset open cavity. In the experiment, a high sensitivity of −17,905 nm/RIU was achieved in the refractive index (RI) measurement. On this basis, the probe DNA (pDNA) was immobilized onto the sensor’s surface using APTES, enabling real-time monitoring of captured complementary DNA (cDNA) samples. The experimental results demonstrate that the biosensor exhibited a high sensitivity of 0.32 nm/fM and a limit of detection of 48.9 aM. Meanwhile, the sensor has highly repeatable and specific performance. This work reports an easy-to-manufacture, ultrasensitive, and label-free DNA biosensor, which has significant potential applications in medical diagnostics, bioengineering, gene identification, environmental science, and other biological fields.

## 1. Introduction

With the continuous development of molecular biology and genetic engineering, DNA hybridization technology has gained immense significance in fields such as gene detection and disease diagnosis. Matching detection of DNA sequences is widely used in various fields, including bioengineering, disease diagnosis, gene identification, and environmental science, due to advancements in biological detection technology [1,2,3,4,5,6]. Currently, chemical methods such as polymerase chain reaction (PCR) and loop-mediated isothermal amplification (LAMP) have been extensively applied in these fields [7,8,9,10]. However, these methods require complex and lengthy processes, resulting in challenges associated with high cost, complexity, and time consumption [11]. Although electrochemical DNA sensor can solve some of these issues [12,13], their susceptibility to environmental interference remains a concern [14].

Optical fiber sensors have recently gained more and more attention because of their ability to serve as label-free, real-time, highly sensitive, and rapidly responsive DNA hybridization sensors. Currently, various optical fiber sensors are available for in situ DNA hybridization detection, including optical fiber surface plasmon resonance (SPR) sensors [14,15,16], optical fiber gratings [11,17,18], and optical fiber interferometers [19,20,21,22]. Leung A et al. reported on gold-coated tapered optical fiber sensors for DNA detection. This sensor was used to detect 750 fM complementary DNA [15]. However, this sensor faced difficulties in distinguishing between low-concentration DNA with high-concentration DNA. Yang et al. proposed a Gr/Ag-coated tilted grating sensor for detecting DNA hybridization [14]. This sensor has high sensitivity, with a limit of detection (LOD) for DNA as low as 3 pM. However, its reliance on tilted gating and SPR makes the fabrication complex, requiring a special polarization light source to excite the SPR effect. Zainuddin et al. proposed a DNA hybridization detection method utilizing a carbon quantum dots-functionalized tapered optical fiber [19]. This sensor achieved an LOD of 1.0 fM and exhibited substantial affinity and high sensitivity of 1.8295 nm/nM. However, the fragile structure of tapered optical fiber sensors raises durability concerns, while the use of carbon quantum dots increases production expenses, thus increasing overall costs. Li et al. developed a C-type optical fiber sensor based on reflective Fabry–Perot interference (FPI) [21]. Although this sensor achieves high sensitivity, it relies on the Vernier effect for sensitivity enhancement, potentially decreasing stability [23] and introducing complexities in signal processing. In addition, optical fiber DNA sensors have limitations in terms of the number of tests. Therefore, rapidly resolving the issues of a high sensitivity, low cost, ease of use, and fabrication of sensors is necessary.

In this paper, we proposed a fiber microcavity Mach–Zehnder interferometer (MZI) sensor for real-time, label-free, and highly sensitive specific detection of DNA hybridization. The proposed fiber biosensor has an open microcavity formed by offset splicing of three sections of standard communication single-mode fibers (SMFs), allowing for the easy introduction of gas or liquid samples into the open cavity [24]. Using such an open cavity structure, higher sensitivity and amplification of the DNA hybridization signal were achieved through the surface functionalization of the MZI sensor. Compared to SPR biosensors and optical fiber gratings, the fiber MZI biosensor offers high sensitivity, reduced cost, and simplified sample fabrication. The microcavity MZI biosensor provides an attractive solution for real-time in situ detection of DNA hybridization. This sensor holds great promise in working with other nucleic acid biotechnology in the future.

## 2. Materials and Methods

### 2.1. Sensing Principle and Fabrication of MZI Sensor

**Figure 1:**
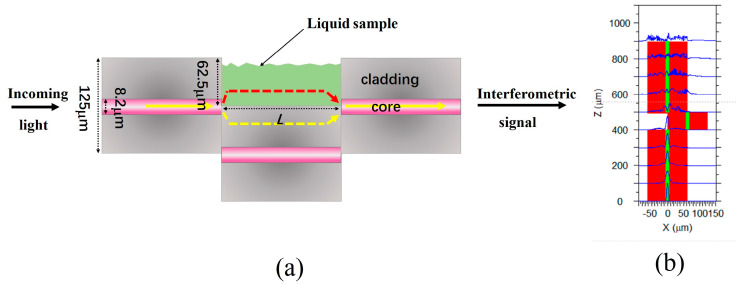
(**a**) The schematic diagram of the open-cavity MZI sensor. (**b**) Mode field distribution in the cross-section of the MZI structure.

A schematic diagram of the open cavity MZI sensor is shown in Figure 1a. There are two large lateral offset splicing points that function as splitting and combining units. These two splicing points are formed by splicing three sections of SMFs. At the first splicing point, the incident light is divided into two parts. One part of the light passes through the cladding of the SMF (depicted as yellow light in Figure 1), whereas the other part propagates through the microcavity (represented by red light in Figure 1a). At the second splicing point, the two parts of the light interfere with each other because they experience different optical paths. Figure 1b shows a simulation of the mode field distribution within the structure by using Rsoft 2018 software. The light is divided into two parts in the first splicing point and then coupled at the second splicing point. This structure belongs to the in-fiber MZI sensor. Since the MZI sensor has an open cavity that can be filled with liquid or gas, it enables the analysis of liquid or gas samples within the microcavity.

According to the interference theory, the transmission intensity of the MZI sensor can be expressed as:

(1) $$I(\lambda )=I_{1}+I_{2}+2\sqrt{I_{1}I_{2}}\cos(\frac{2\pi L(n-n_{cl})}{\lambda }+\phi_{0})$$

where *I*_1_ and *I*_2_ represent the light intensity along the cladding of the SMF’s and MZI’s open cavity, respectively. λ denotes the free space wavelength in vacuum, *L* is the length of MZI cavity, *n* is the refractive index (RI) of the space in the MZI cavity, *n_cl_* is the RI of the SMF cladding, and $\phi_{0}$ represents the initial interference phase. According to Equation (1), the valley in the interference wave within the transmission spectrum indicates that the interference phase is an odd multiple of π.

When the RI of the sample in the MZI cavity changes from *n* to *n +* Δ*n*, the wavelength of the interference valley shifts accordingly. Considering the wavelength shift of the *m*th order interference valley $\lambda_{m}$, it shifts to $\lambda_{m}$ *+* $\Delta \lambda_{m}$. Since the phase value of the cosine term keeps as (2*m* + 1)π, we have:

(2) $$\frac{2\pi (n_{cl}-n)L}{\lambda_{m}}+\phi_{0}=\frac{2\pi [n_{cl}-(n+\Delta n)]L}{\lambda_{m}+\Delta \lambda_{m}}+\phi_{0}=\left(2m+1\right)\pi$$

After simplification, the RI sensitivity of the biosensor can be expressed as the following equation:

(3) $$S=\frac{\Delta \lambda_{m}}{\Delta n}=-\frac{\lambda_{m}}{n_{cl}-n_{}}$$

where *m* is an integer and $\lambda_{m}$ is the wavelength of *m*th order interference valley. Equation (3) illustrates that the wavelength of the interference valley exhibits a blue shift if the RI of the MZI cavity increases, while a decrease in the RI causes a red shift. According to Equation (3), the RI sensitivity of the MZI sensor depends on the original wavelength of the valley. The longer the referenced wavelength, the higher the RI sensitivity of the MZI sensor.

The wavelength interval between two adjacent valleys in a transmission spectrum is called the free spectral range (*FSR*), and it is given by:

(4) $$FSR=\frac{\lambda_{m}\lambda_{m+1}}{(n_{cl}-n)L}$$

where $\lambda_{m+1}$ is the wavelength of (*m* + 1)th order interference dip. Equation (4) shows that the length of the microcavity affects the range of *FSR* in the transmission spectrum and the wavelength position of the transmission dip. The longer the cavity length, the smaller the *FSR* of the interferometer, the denser the interference fringes. When the interference fringes are dense, the spectral width of transmission loss peak is narrow, which is beneficial to improving the detection resolution.

The MZI sensor was fabricated using standard SMFs (Coring SMF-28) and a commercial fusion splicer (Fujikera FSM-100P, Tokyo, Japan). The MZI sensor was fabricated in four steps: First, two pieces of SMF were cleaved to create interfaces without any angles. They were then placed into the fiber splicer with modified parameters. In this spicing mode, the discharge current and time are set as 206 bits and 1900 ms, and the offset parameter of X-direction is set as 62.5 μm. After splicing the first point, the fiber was moved under a microscope and the offset SMF with a desired cavity length was cleaved. The MZI cavity length was set to several hundred micrometers to form an open-cavity structure. The length of the microcavity affects the range of FSR in the transmission spectrum and the wavelength position of free transmission dip. Utilizing automated equipment allows for precise adjustment of open-cavity length. Finally, the end-face of the offset SMF was spliced into another cleaved SMF in the splicer in manual operation mode. The modified parameters included a discharge current of 210 bits, a discharge time of 2000 ms. During the splicing process of the second offset point, the manual operation mode was used. The two fibers were manually adjusted to ensure no offset in the Y-direction, and the lateral offset in the X-direction is approximately 62.5 μm. In this experiment, we try to achieve maximum contrast of interference fringes by manually adjusting the offset value in the X-direction of the second splicing point. By maximizing the contrast of interference fringes, we can reduce experimental errors and improve the repeatability of the sensor through active alignment. The active alignment means the offset parameter was adjusted by maximizing the contrast of interference fringes through real-time monitoring of an optical spectrum analyzer (OSA, YOKOGAWA AQ6370D, Tokyo, Japan).

### 2.2. Materials

The materials used in the experiment are as follows:

- NaOH (0.1 M; Sigma), 3-aminopropyl triethoxysilane (APTES 10% *v*/*v*; Cool Chemistry), TE buffer, pure water, and anhydrous ethanol.
- In this work, DNA was purchased from Sangon and these sequences are as follows:
- Proble DNA (pDNA):
- 5′-AGGAGGAGACTTAAGTAAAA-3′ (5′ was modified by carboxy (-COOH));
- Complementary DNA (cDNA):
- 5′-TTTTACTTAAGTCTCCTCCT-3′;
- Non-complementary DNA (nonDNA):
- 5′-CTCACGTTAATGCATTTTGGTC-3′;

The DNA samples solution were prepared with pure water and saved at −20 °C.

### 2.3. Experimental System

**Figure 2:**
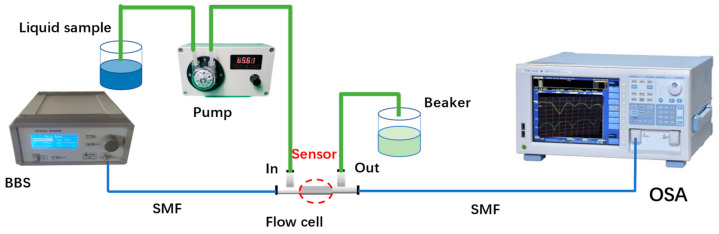
The DNA detection experiment system based on the MZI sensor.

The DNA detection system based on an open-cavity MZI biosensor is illustrated in Figure 2. The fiber MZI sensor was connected to a broadband light source (BBS), which provided a wavelength range of 1200 nm to 1700 nm. The other end of the fiber MZI sensor was connected to an optical spectrum analyzer (OSA). The OSA (YOKOGAWA AQ6370D, Tokyo, Japan) with a wavelength resolution of 0.02 nm was used to monitor the interference transmission spectrum of the microcavity MZI. In the experiment, the sensor was securely mounted within a liquid channel created by two polypropylene ‘T’ shape channels (channel parameters: inner diameter: 1.4 mm; outer diameter: 2.3 mm; length: 23.4 mm; width: 14.2 mm) and a silicone tube (parameters: inner diameter: 2 mm; outer diameter: 4 mm). When inserting the sensor into the T shape channel, UV glue was used to fill the horizontal ports, while the vertical ports were connected to the pump (Lianhezhongwei Company LHZW005-3LG0.4×3, Beijing, China) via a silicone tube. To ensure that the fiber is straight in the T shape channel, some stress should be applied on the fiber when the sensor is packaged by use of UV glue. If the fiber is bent, the interference fringe contrast of the transmission spectrum deteriorates, which affects the performance of the sensor.

## 3. Results and Discussion

### 3.1. RI Sensitivity Measurement of the MZI Sensor

**Figure 3:**
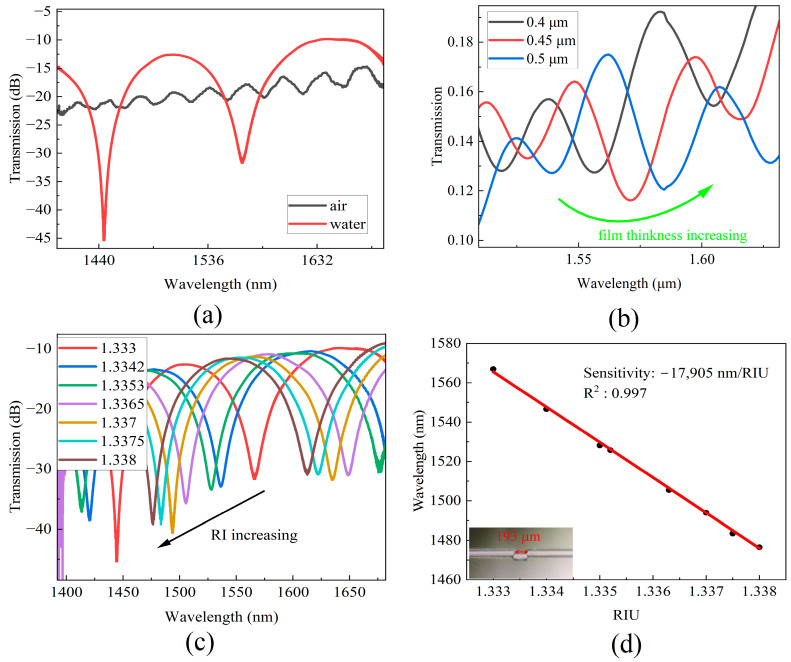
(**a**) Transmission spectra in air and water. (**b**) Simulated transmission spectra change with film thickness of MZI cavity increasing. (**c**) Transmission spectra at different RI. (**d**) The wavelength shifts of MZI correspond to RI and the photo of MZI sensor.

A fiber MZI with a cavity length of 193 μm was fabricated according to the steps described in Section 2. The transmission spectra of the MZI sensor in air and water are depicted in Figure 3a. When the MZI was placed into a liquid environment, the transmission spectrum exhibited an interference fringe contrast of approximately 20 dB, which is suitable for molecular biology detection. The DNA detection process involves two mechanisms that contribute to the MZI response. On one hand, an increase in the refractive index (RI) at the bottom of the cavity impacts the propagation of light within the cladding (*n_cl_*), resulting in a red shift of the spectra [25]. When a cavity is coated with a film through chemical functionalization or DNA deposition, the RI of the cavity surface also increases [22]. 

To investigate the influence of the film thickness on the MZI response, simulations were performed using Rsoft to examine the relationship between the spectra and film thickness. The RI of the film material was set to 1.500, and film thicknesses of 0.4 μm, 0.45 μm, and 0.5 μm were simulated. Figure 3b illustrates the impact of film thickness on the transmission spectra, where an increase in the thickness of the thin film results in a shift of the interference minimum wavelength towards the longer wavelength region.

On the other hand, the RI of the ambient medium affects the propagation of light in the cavity. In the open cavity of the MZI structure, a fraction of the light passes directly through the microcavity and is affected by the RI of the liquid contained within, since the two parts of light in the open cavity and in the fiber cladding experience different optical path. At the second splicing point, the portion of light in the open cavity then interacts with the light that propagates in the cladding of the microcavity structure. Consequently, any variations in the RI of the liquid sample inside the microcavity immediately induce the wavelength shift of the transmission spectrum. 

To investigate the RI response of the MZI sensor, RI sensitivity measurements were performed using solutions of water and alcohol in different proportions. Because the refractive index of the DNA liquid sample was approximately 1.3336, the RI of the test samples varied in the range from 1.3330 to 1.3380. In each measurement, a predetermined amount of alcohol is introduced into the water, and the transmission spectrum is recorded until it reaches a stable state. Subsequently, a sample adjacent to the microcavity is extracted, and the RI of the liquid sample is measured using an Abbe refractometer. 

Figure 3c shows the transmission spectra at different RI levels. It is evident that the transmission interference dips shifted towards to the short wavelength region as the RI of the sample increases. The wavelength of 1560 nm was selected as the monitored wavelength. Figure 3d shows the wavelength shift with an increase in the sample’s RI. Linear fitting of the experimental data was performed, and a wavelength sensitivity of −17,905 nm/RIU was obtained with a linear correlation coefficient of R^2^ = 0.997.

The figure of merit (*FOM*) is also an important parameter that evaluates the capability to sensitively measure changes within a tiny wavelength [26,27,28]. In this work, it can be described as follows [28]:

(5) $$FOM=\frac{\lambda_{0}}{\lambda_{FWHM}}=\frac{1560\;\mathrm{nm}}{19.6\;\mathrm{nm}}=79.6$$

where $\lambda_{0}$ is the wavelength dip of the microcavity MZI spectrum and $\lambda_{FWHM}$ is the full width at half minimum for the interferometer spectrum. In this experiment, $\lambda_{0}$ = 1560 nm, $\lambda_{FWHM}$ = 19.6 nm.

### 3.2. Results of Complementary DNA Hybridization Detection

**Figure 4:**
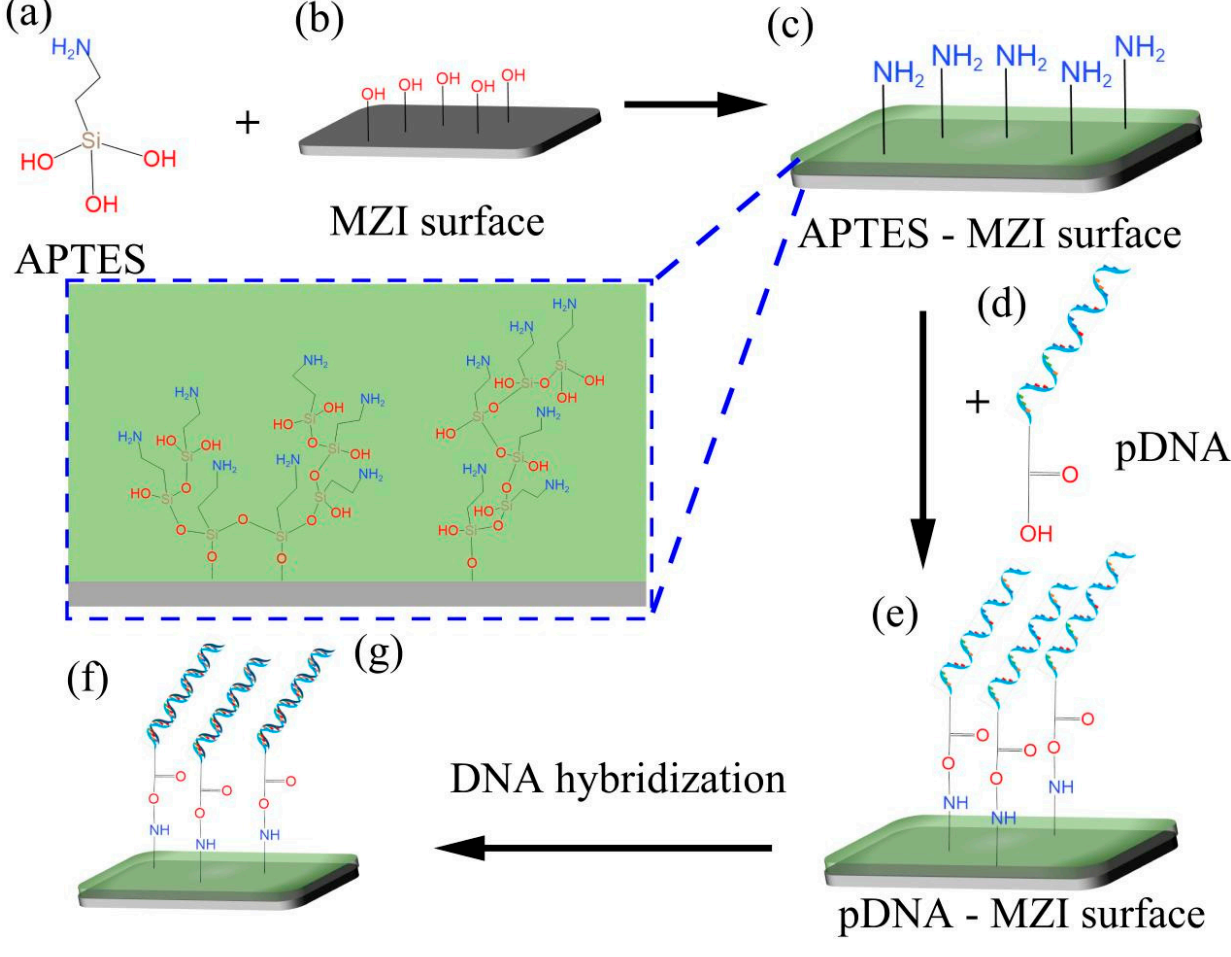
The process of the MZI’s surface functionalization. (**a**) APTES molecular formula. (**b**) MZI surface with hydroxylation. (**c**) Amino-functionalization surface of sensor. (**d**) Structure of pDNA. (**e**) pDNA linked to the surface using APTES as crosslinker. (**f**) DNA hybridization on surface of sensor. (**g**) Schematic diagram of the linker modified onto the sensor’s surface.

The functionalization process plays a crucial role in linking biomolecules and inorganic materials to the surfaces of MZI sensors. This process enables DNA hybridization to occur on the sensor’s surface, as shown in Figure 4. In this experiment, APTES (its molecular formula shown in Figure 4a) was used as the crosslinker. Before surface functionalization, the MZI’s surface was cleaned by using a mixture of pure water and ethanol. To activate the hydroxyl groups (-OH) and remove any residual chemicals on the surface, a 0.1 M NaOH solution was pumped into the liquid channel for 50 min [29,30]. After rinsing with pure water and drying at room temperature, the sensor’s surface exhibited reactive hydroxyl groups [31], and the schematic diagram of this process is shown in Figure 4b. Following this initial step, the sensor was immersed in 10% APTES solution for 50 min. APTES contains Si-OH and -NH_2_ in its molecular structure, and Si-OH can react with -OH on the MZI’s surface to become Si-O-Si via dehydration [32,33], as shown in Figure 4g. Consequently, APTES converts hydroxyl groups into amine groups, enabling the sensor’s surface to bond with carboxy groups (-COOH), as depicted in Figure 4c. After functionalization process of the sensor’s surface, the sensor was thoroughly washed with pure water and TE buffer to remove excess APTES molecules. Subsequently, 10 nM pDNA solution was injected into the flow cell for 50 min, as illustrated in Figure 4d. In this process, pDNA was modified with carboxy groups (-COOH) [20,21], facilitating its immobilization on the sensor’s surface through NH_2_-COOH bonding, as indicated in Figure 4e. Figure 4f shows a schematic diagram of DNA hybridization on the surface of the sensor, when cDNA solution was injected into the flow cell.

To evaluate the sensing response of DNA hybridization, a series of cDNA concentrations (1 fM and 10 fM) were pumped into the liquid channel for 15 min for experimental testing. Between each test, an 80 °C pure water solution was pumped to denature the double-stranded DNA, ensuring the dissociation of cDNA and allowing for consistent pDNA numbers on the MZI’s surface. 

**Figure 5:**
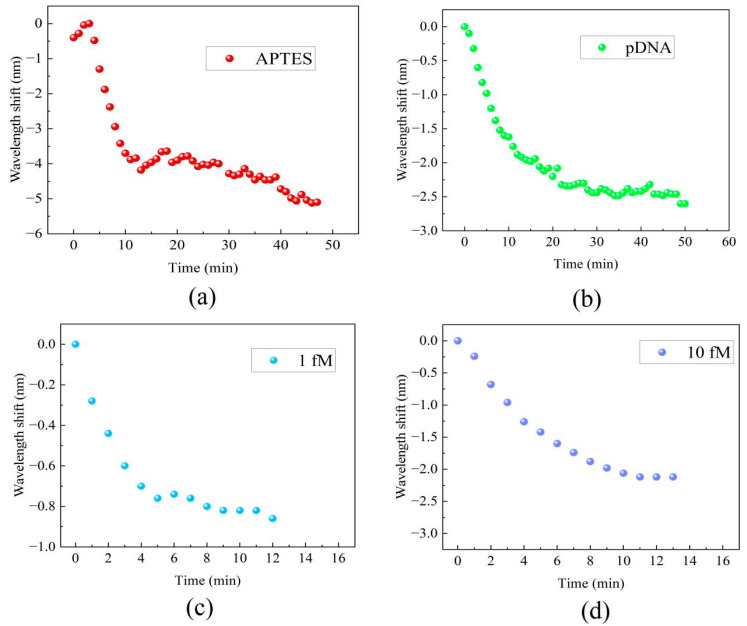
The real-time wavelength shift responses of the MZI: (**a**) the functionalization of APTES, (**b**) immobilization of pDNA, (**c**) cDNA hybridization at a concentration of 1 fM, (**d**) DNA hybridization at a concentration of 10 fM.

Real-time monitoring of the transmission spectral responses was performed to track the processes of the MZI’s surface modification, functionalization, and detection of DNA hybridization at various concentrations. Figure 5 displays the real-time wavelength shift responses of the MZI during its surface functionalization and DNA hybridization. The interaction between the MZI’s surface and APTES amino groups resulted in alterations in the film thickness on the cavity surface and concurrent changes in the RI within the cavity. The wavelength shift during the functionalization process of APTES is shown in Figure 5a. The initial red shift about 0.5 nm in wavelength observed in Figure 5a during APTES functionalization was attributed to the increase in film thickness on the MZI’s surface, while the gradual blue shift of the spectrum was caused by the increased RI on the cavity surface. During the process, the interference spectra exhibited a wavelength shift of 5.1 nm towards the short-wavelength region. This indicates that the MZI sensor possesses an ultrasensitive response to APTES. As the result, it is apparent that the influence of coating patterns on sensor performance is considerably smaller in comparison to the impact caused by changes in the surface refractive index. When pDNA was introduced into the liquid channel, the strong absorption interaction between the APTES layer and the carboxy groups of pDNA resulted in wavelength shifts of up to 2.6 nm, as shown in Figure 5b. After pDNA was immobilized on the MZI’s surface, 1 fM cDNA and 10 fM cDNA were introduced into the liquid channel. The complementary DNA (cDNA) undergoes hybridization, forming a stable double helix structure when paired with the probe DNA. The hybridization interaction alters the refractive index of the microcavity’s surface. Due to the complementary base pairing, wavelength shifts of 0.86 nm and 2.12 nm were observed within just 10 min, and the wavelength shifts are shown in Figure 5c and Figure 5d, respectively.

**Figure 6:**
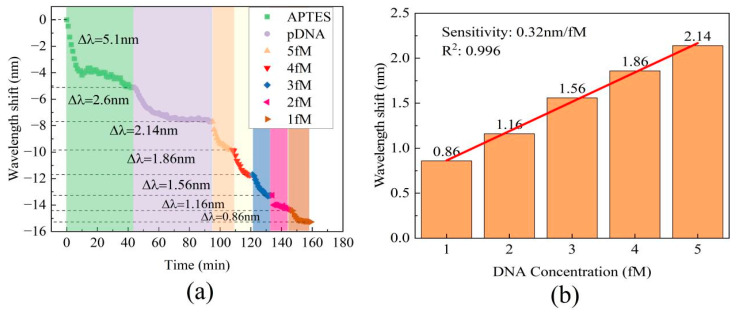
(**a**) The real-time wavelength shift of the MZI in the processes of surface functionalization and DNA hybridization. (**b**) The absolute value of the wavelength shift at various DNA concentrations.

To evaluate DNA hybridization detection capability, different cDNA samples with concentrations ranging from 2 fM to 5 fM were injected into the liquid channel within just 10 min. After each measurement, it is recommended to rinse the sensor’s surface with 80 °C pure water. This step facilitates the denaturation of the double-stranded DNA; thus, it guarantees that the amount of probe DNA on the sensor remains consistent prior to each subsequent detection. All cDNA samples resulted in wavelength shifts towards shorter wavelengths, as depicted in Figure 6a. The relationship between cDNA concentration and wavelength shift in the transmission spectra followed a linear trend, as demonstrated in Figure 6b. This result indicates a wavelength shift of 2.14 nm for the 5 fM sample, which is similar to the wavelength shift of 2.12 nm observed for the 10 fM sample. This similarity could be attributed to the limited number of pDNA molecules on the cavity surface. The range of cDNA concentrations covered in the detection interval is from 1 fM to 5 fM, with a sensitivity of 0.32 nm/fM and high linearity of R^2^ = 0.996.

**Figure 7:**
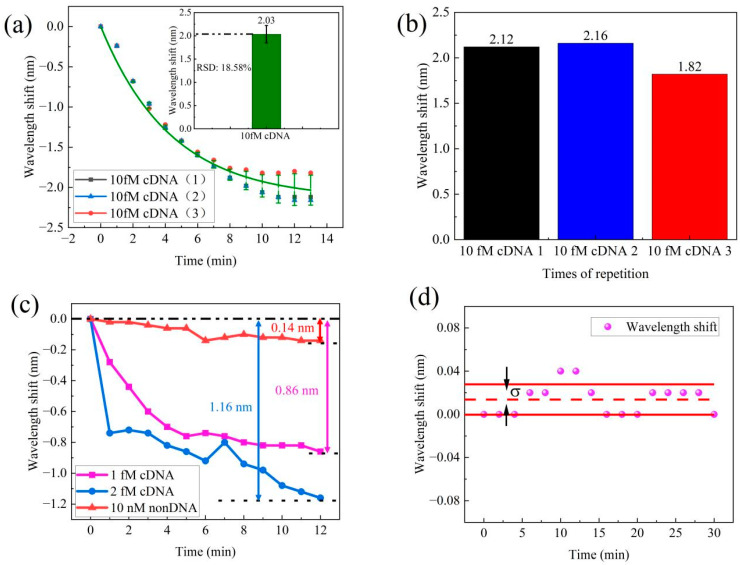
(**a**) Repeatability test of the MZI for 10 fM cDNA samples. (**b**) The spectra wavelength shift of repeatability tests. (**c**) Control test for non-DNA of the MZI sensor. (**d**) Stability test.

In order to assess the repeatability of DNA hybridization detection, we conducted three repetitions of the experiments using a concentration of 10 fM cDNA. The cDNA solution was pumped into flow cell and we recorded the transmission spectra for 10 min. After performing three repetitions of the process, we compared data from each process to verify the sensor’s repeatability. The corresponding wavelength shifts are shown in Figure 7a. The results indicate an average wavelength shift of 2.03 nm, with a relative standard deviation (RSD) of 18.5%. Figure 7b presents the results of the three repeated tests, which resulted in wavelength shifts of 2.12 nm, 2.16 nm, and 1.82 nm. In conclusion, this sensor demonstrated excellent repeatability, hence making it suitable for practical applications.

To evaluate the specificity of the MZI sensor, a control experiment was performed using 10 nM non-DNA solution. We recorded the wavelength shift data within 10 min. The transmission exhibited different wavelength shifts for cDNA and non-DNA solution. The spectral wavelength data for this experiment are presented in Figure 7c. In this study, cDNA solutions at concentrations of 1 fM and 2 fM were examined, both resulting in wavelength shifts towards shorter wavelengths. Specifically, the 1 fM cDNA solution displayed a 0.86 nm wavelength shift, while the 2 fM cDNA solution exhibited a slightly larger wavelength shift of 1.16 nm. In contrast, the 10 nM non-DNA solution exhibited only a small fluctuation in wavelength, measuring a mere 0.14 nm for the same transmission dip. This difference in magnitude of wavelength shifts between the cDNA solutions and the non-DNA solution clearly illustrates the high specificity of our MZI sensor for detecting DNA hybridization. This result also proves the sensitivity of DNA detection is from DNA hybridization between pDNA and cDNA, and the low concentration of DNA is difficult to distinguish using the RI of solution. 

The detection limit is defined as the minimum concentration or amount of the analyte in a sample that can be accurately differentiated from zero [34]. To assess the stability of the sensor, a 30 min test was conducted, with wavelength shift measurements taken every 2 min, as depicted in Figure 7d. Standard deviation was utilized to evaluate the extent of potential changes or fluctuations within this dataset. The smaller the standard deviation, the narrower the range of the data fluctuations, indicating greater stability. The standard deviation of the wavelength shift of the MZI sensor was measured as 0.014 nm. These results confirm that the MZI sensor exhibits excellent stability and is promising for practical applications. By utilizing this standard deviation measurement in the sensor response, the detection limit of the MZI sensor can be estimated. According to the International Union of Pure and Applied Chemistry (IUPAC) [35], the limit of detection (*LOD*) was calculated by dividing three times the signal standard deviation of the blank sample by the sensitivity of the sample, as described in Equation (7).

(6) $$LOD=\frac{3\sigma }{S}$$

where σ represents the standard deviation of the blank sensor sample during the steady state and S is the RI sensitivity. Consequently, we can determine the *LOD* for the liquid RI using the experimental data. The *LOD* of the RI obtained was 2.3 × 10^−6^ RIU. During the DNA sensing experiment, the RI of the sensor’s surface underwent a change of 4.7 × 10^−5^ RIU when the DNA concentration was set at 1 fM. Therefore, the *LOD* for DNA concentration was calculated as [21]:

(7) $$\frac{2.3\times 10^{-6}\;\mathrm{RIU}}{4.7\times 10^{-5}\;\mathrm{RIU}}\times 1\;\mathrm{fM}=48.9\;\mathrm{aM}$$

Quality factor is also one of the main parameters for biosensor. For this MZI biosensor, the quality factor can be expressed by the following equation [36]:

(8) $$Q=\frac{\lambda_{0}}{10\times \Delta \lambda_{min}}\approx \frac{1560\;\mathrm{nm}}{10\times 0.02\;\mathrm{nm}}=7300$$

where $\lambda_{0}$ is the wavelength dip of the MZI spectrum and $\Delta \lambda_{min}$ is minimal resolvable wavelength shift. In this experiment, the value of $\Delta \lambda_{min}$ is 0.02 nm, that is, the wavelength resolution of the OSA.

The fiber microcavity biosensor successfully achieved label-free, in situ, and real-time DNA hybridization detection with a notably low detection limit and high specificity. Table 1 shows the experimental results of our sensors compared with previous works. It can be seen that our microcavity MZI biosensor has higher RI sensitivity than other free-label biosensors. In particular, the RI sensitivity of our sensor is 10 times of that in Ref. [20]. Furthermore, the *LOD* of our biosensor is much smaller than that of in previous works [19,20,21,37]. The proposed SMF microcavity biosensor has the advantages of simple structure, low cost, and high sensitivity; it has a broad application prospect in the field of biosensors.

## 4. Conclusions

In summary, we proposed a highly sensitive, low-cost, and label-free fiber micro-cavity biosensor for specific detection of DNA hybridization. The MZI sensor was fabricated just using large-offset splicing SMFs. The proposed sensor achieved an ultrahigh sensitivity of −17,905 nm/RIU for RI detection. The immobilization of pDNA on the surface of the MZI sensor facilitated using APTES as a crosslinker allows for the selective capture of cDNA samples. Our experimental results demonstrate that the fiber microcavity biosensor is capable of monitoring real-time DNA binding processes with high sensitivity. The proposed microcavity MZI biosensor can detect cDNA within a linear range from 1 fM to 5 fM, with a sensitivity of 0.32 nm/fM, high linearity of 0.996, and impressive detection limit of 48.9 aM. The simple structure and cost-effective manufacturing of the label-free biosensor make it a promising candidate for various applications in medical diagnostics, gene identification, environmental science, and other domains within biological fields.

## Figures and Tables

**Table 1:** Comparison of sensing performance between different DNA sensors.

Structure	RI Sensitivity	LOD	Ref.
Tapered optical fiber	2235 nm/RIU	1 fM	[19]
ECF + MZI	1771 nm/RIU	0.31 nM	[20]
C-type fiber + SMF	10,791 nm/RIU	67.5 nM	[21]
Microfiber-assisted MZI	−11,571 nm/RIU	0.0001 pmol/μL	[37]
SMF + MZI	−17,905 nm/RIU	48.9 aM	Our work

## Data Availability

Data are contained within the article.
